# Evaluation of the Effect of Thermo-Oxidative Aging and UV Radiation on Asphalt Stiffness

**DOI:** 10.3390/ma16103716

**Published:** 2023-05-13

**Authors:** Eva Remisova, Dusan Briliak

**Affiliations:** Department of Highway and Environmental Engineering, Faculty of Civil Engineering, University of Zilina, Univerzitna 8215/1, 010 26 Zilina, Slovakia; briliak1@stud.uniza.sk

**Keywords:** asphalt, thermo-oxidative aging, UV radiation, stiffness, indirect tensile strength, ageing index, climatic factors

## Abstract

The complex factors during the asphalt production process and subsequent traffic loading, climatic and weather conditions lead to a decrease in durability of asphalt thereby reducing pavement surface service life. The research focused on the effect of thermo-oxidative aging (short and long term), ultraviolet radiation and water on stiffness and indirect tensile strength of asphalt mixtures with 50/70 and PMB45/80-75 bitumen. The stiffness modulus at different temperatures (10, 20 and 30 °C) using the indirect tension method and indirect tensile strength have been evaluated in relation to the degree of aging. The experimental analysis showed a significant increase in the stiffness of polymer-modified asphalt with increasing aging intensity. Exposure to ultraviolet radiation adds to an increase of 35 to 40% in stiffness for unaged PMB asphalt and 12 to 17% for short-term aged mixtures. Accelerated water conditioning reduced the indirect tensile strength of asphalt by an average of 7 to 8%, significant in long-term aged samples using the loose mixture method (9 to 17%). The indirect tensile strengths for dry and wet conditioning showed greater changes due to the degree of aging. An understanding of the changes in asphalt properties during design allows for predicting asphalt surface behaviour after a period of use.

## 1. Introduction

Trends in pavement design focus on long-life and durable pavements and durability (durable enough to resist traffic and the environment) while ensuring structural integrity and thickness to prevent deterioration such as fatigue cracking and permanent deformation [1], following the concept of Forever Open Road for road construction and maintenance, applied on the highway, rural or urban roads and regardless of region or country by FEHRL (Forum of European National Highway Research Laboratories).

For pavements in this way, the durability of the surface layer is the dominant factor for the service life. Wearing course performance requirements include resistance to rutting and surface cracking, ensuring adequate skid resistance, permeability, noise reduction and wear resistance [2].

The complex climatic and weather conditions and time results in the aging of materials due to conditions during asphalt mixture production, transport and paving as well as in service life. These processes affect the mixture properties in the short term of a few hours, referred to as short-term aging (STA), and in the long-term stage that occurs for a few years while the asphalt surface layer is in service as long-term aging (LTA) [3]. The factors such as heat, oxygen, solar radiation with ultraviolet radiation part, water and maintenance agents used in winter are significant in terms of influence. Endogenous factors include the composition of asphalt material, binder content and related thickness of binder film on the aggregate surface, composition and properties of bitumen itself and performance properties to bond mineral aggregate and air void content [4,5]. Aging reduces the long-term performance of asphalt mixtures and makes the binder and asphalt mixture brittle and stiff to harden [6].

Aging is a very complex process. According to [7], bituminous binders are susceptible to hardening and after-aging processing. The hydrocarbons in bitumen react with oxygen, the contents of oxygen containing functional groups such as carbonyl and sulfoxide that increase, and the weight of molecules in bitumen also increases. The main reasons for bitumen aging are the loss of light components and oxidation reactions. The indicators of bitumen properties change, penetration decrease, softening point, stiffness and viscosity increase, leading to failure in the field as stated in [8,9,10].

Significant changes occur in the asphalt mix production processes (mixing, transport and paving) when the bitumen binder is heated to a high temperature (hot asphalt processing), an oxidation reaction takes (the components of the bitumen binder oxidize to form heavier and more complex molecules) and volatilization of the lighter oil fractions (decrease in maltenes content due to volatilization, volatilization is mostly a function of temperature, increase with temperature elevating) [11]. In terms of bitumen composition, the contents of saturates and aromatics (light components) decrease, and the contents of resins and asphaltenes extend. In the production of the asphalt mixture [12], the oily components of the bitumen migrate into the aggregate, depending on the composition and porosity of the aggregate, these migration processes are referred to as exudation.

The effects ageing, thus oxidation reaction and volatilization of the light fractions, increase the molecular weight of the bitumen and intermolecular bond strength. This is then reflected in changes in the physical properties, rheological properties of the bitumen, changes in the chemical composition (functional groups), surface morphology, etc. [13,14].

The intensity of volatilization ageing is mainly influenced by temperature and depends on the exposure of the bitumen to high temperatures (asphalt production, transport and laying). During service, the bitumen in the mixture is subjected to weathering environmental conditions (temperature, moisture), in particular in the bitumen reaction with oxygen [15]. The oxidation reaction further consumes the oxygen diffused in the bitumen, leading to further oxygen diffusion. In addition, aging can deteriorate the colloidal structure in which the asphaltenes act as micelles surrounded by resins and change their physico-chemical properties. Water and moisture accelerated asphalt aging, water can diffuse through the bitumen and cause a reduction in cohesion. Moisture can further achieve the aggregate surface and lead to adhesion damage and stripping. The diffusion process is generally followed by the Fick rule but bonding interaction between a water molecule and functional groups of bitumen [16,17]. Results in studies [18,19] have shown that moisture and temperature can disturb the colloidal structure of bitumen (chemical aging) and thus affect its rheological properties but have no significant effect on the rate of oxidative ageing itself.

Sunlight initiates the degradation process that interacts with heat and moisture to cause adverse effects. Although the effect of solar radiation on bitumen has been known, the influence of ultraviolet radiation on bitumen binders and asphalt surfaces is often neglected but UV radiation act on the top layers of the pavement surface. UV radiation activates a photo-oxidation reaction in bitumen, penetrating a thickness of 4–5 μm. The oxidized bitumen is water soluble and can be removed, exposing fresh unaged bitumen. The photo-oxidation reaction is also supported by the presence of a constant supply of fresh oxygen and occasional high temperatures on the road surface. The depth of UV ageing in bitumen gradually increases with increasing UV ageing time, which is explained by the ‘diffusion ageing model’ [20]. In the case of UV radiation, the effect is limited to the surface layer only, therefore UV ageing should not be ignored in the case of pavement surfacings. The rate of ageing decreases gradually over the course of long-term ageing, with the most pronounced ageing period being the first two to three years. Studies [21,22,23] investigating the effect of UV radiation have been realized; however, under different conditions, since there is no unified standard to perform UV ageing experiments. UV ageing methods used by different researchers are quite different and the effect of test conditioning depends on the UV radiation intensity, simultaneously applied temperature and also bitumen film thickness.

Termoreversible ageing, besides oxidation, volatilization, ultraviolet aging, and exudation, is an important factor leading to early cracking in asphalt pavement. It develops when the bitumen binder is exposed to low temperatures or middle temperatures for a prolonged period of time. It is also referred to as physical ageing. It is the reorientation and fixation of the arrangement of atoms in a molecule [24]. As a result of the change in the orientation of the molecules, physical ageing changes the rheological properties of the bitumen. It can appear so that the measured value of bitumen property, e.g., the stiffness modulus, is dependent on the thermal history of the sample. This process is reversible because it changes the physical properties of the bitumen without changing its chemical composition.

Laboratory methods for simulating the thermal ageing of bitumen binders are standardized. The aim is to achieve the same degree of ageing of bitumen in laboratory conditions and use during construction and in service. Laboratory ageing methods use the principle of time–temperature dependence, where the real ageing process at lower temperatures for longer periods of time can be replaced by ageing at higher temperatures and for shorter periods of time. This means that elevated temperatures, thin bitumen film thickness or pressure are used [25]. Bitumen short-term aging is simulated in the laboratory conditions using different methods, such as the Rolling Thin Film Oven Test (RTFOT), performed according to EN 12607-1 or Thin Film Oven Test. For long-term aging, bitumen is processed using a pressure aging vessel (PAV) or multiple RTFOT test. Using various simulation techniques, it is able to understand bitumen aging behaviour and predict its performance in asphalt courses and road pavements [26,27,28]. 

Most laboratory ageing studies only focus on the laboratory ageing of bitumen binders and the search for the relationship between the chemical and rheological properties of aged binders. There is a need to clarify the influence of other asphalt material components, such as aggregate, on the aging of bitumen during the aging process of asphalt mixtures. It is crucial to understand the aging mechanism of asphalt surfaces to improve the service life of asphalt pavement. Understanding the aging process of asphalt mixtures could lead to better design and more appropriate material selection to increase performance and extend the service life of the road pavement.

## 2. Materials and Methods

### 2.1. Test Methods

The objective of laboratory conditioning is to reproduce the degradation processes that occur naturally in a laboratory under accelerated conditions. The laboratory simulation of weathering within days or weeks replaces conditions that occur naturally over the course of months or years in service. The purpose of this study is to evaluate the different ageing factors on asphalt mixture properties. The research was separated into two phases. First, the conditioning in terms of oxidative ageing was applied on asphalt mixtures to the superposed impact of conditions during mixture production and paving (oxidation at higher temperatures) and environmental conditions during service (oxidation, UV radiation and moisture). In the second phase, the stiffness and strength of aged, tested asphalt mixtures were performed and evaluated as a function of the time of aging (see Table 1).

#### 2.1.1. Short-Term and Long-Term Aging

**Figure 1:**
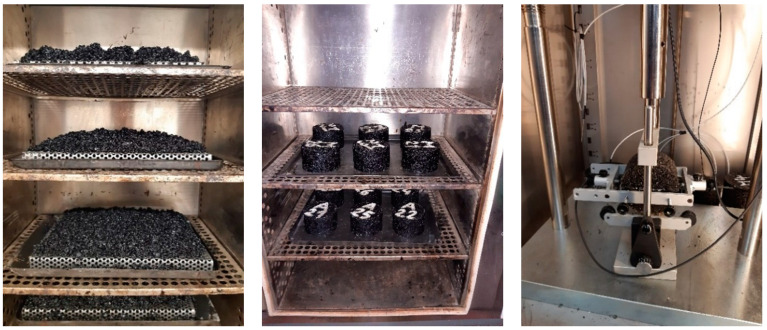
Conditioning of asphalt mixtures at the perforate plate, conditioning of asphalt compacted specimens and stiffness test set with specimen.

In laboratory conditions, the asphalt mixtures were subjected to oxidative aging (according to EN 12697-52 [29]) using a procedure with a loose asphalt mixture and compacted asphalt mixture specimen. Conditioning for the short-term ageing potential involves a loose mixture at the homogeneous layer exposure conditioning of heat and air at a temperature of 135 °C for 4 h following SHRP-A-383. Conditioning for long-term aging potential further loose mixture maintains uniform conditioning at 80 °C for 96 h at the perforated plate (Figure 1) and asphalt compaction to specimens and conditioning at ambient air temperature 85 °C for 120 h.

#### 2.1.2. UV Radiation Exposure

**Figure 2:**
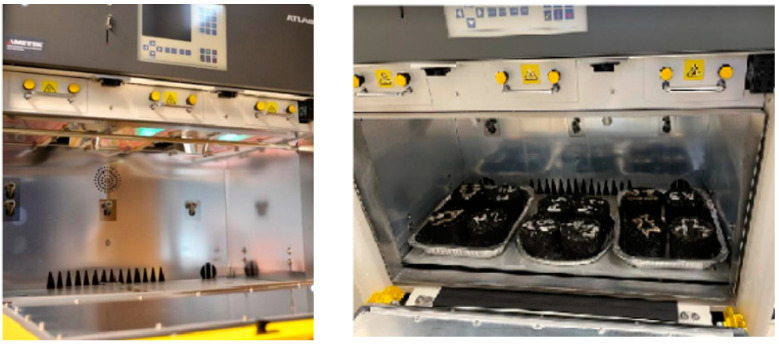
Atlas SUNTEST XXL with test specimens.

Asphalts built in the wearing layer of road pavements are exposed to the effect of solar radiation during service life. It is usual practice in artificial weathering to consider not the total solar radiation energy but the part falling in the ultraviolet region. The UV weathering apparatus Atlas SUNTEST XXL+ (Figure 2) was used for the artificial weathering of asphalt specimens (according to technical report EOTA TR-010 and EN 1297 for bitumen roof sheets). Exposure conditions consisted of radiation and exposure time using xenon light, which is most similar to natural sunlight with a daylight filter with a spectral irradiance of 300 nm to 400 nm (power distribution in Table 2) and the black standard temperature was 60 °C. The UV exposure was carried out up to 600 h and the properties of asphalt mixtures were analysed at intervals of 200 h. 

#### 2.1.3. Stiffness Modulus

To understand the effect of environmental conditions on asphalt mixture properties, the stiffness of the mixture on the cylindrical specimens in a diameter of 100 mm and a height of 30 to 75 mm was evaluated by the IT-CY method (according to EN 12697-26 [30]). The pulse type of loading is applied to the specimen to produce a transient horizontal deformation in the range of 0.005% in the diameter of the specimen. The deformation is measured in a plane perpendicular to the plane of loading. The value of peak deformation shall be 5 microstrains. Loading is characterized by the rise time of 124 ± 4 ms and a pulse repetition period of the applied load of 3 s. The tests were carried out at temperatures of 10, 20 and 30 °C. The calculation is based on assumption that the material is assumed to be homogeneous and isotropic and is loaded in a stress region where it reacts linearly and elastically. Measurement is necessary to achieve a 10% agreement and a confidence interval of 80% with respect to the ‘true value’.

#### 2.1.4. Indirect Tensile Strength

The effect of heat and oxidative ageing was investigated as it affects the indirect tensile strength of the asphalt specimens conditioned in water conditions (according to EN 12697-23 [31] and EN 12697-12 [32]). Non-aged and aged specimens (short-term and long-term aged) were allowed to soak in water and conditioned at an elevated temperature of +40 °C for 68 to 72 h. The test specimens conditioned at a uniform test temperature of +20 °C, aligned between the loading strips and were compression loaded applying a diametrical load continuously at a constant speed of deformation until the specimen breaks. From the peak load, an indirect tensile strength was calculated.

### 2.2. Asphalt Materials

The asphalt mixture studied was asphalt concrete AC11 usually used in wearing layer of flexible pavements for motorways and regional roads in Slovakia. The coarse aggregate (andesite rock from Alas Slovakia s.r.o. quarry), fine aggregate (limestone rock from Dolvap s.r.o., Slovakia) and filler (very finely ground limestone) were used in tested mixtures. The particle size distribution in the aggregate mixture is shown in Table 3. The aggregate used to prepare the asphalts was obtained from plant and asphalt mixtures that were produced in the laboratory following the design parameters according to the type test with a target air void content of 3.0% and bitumen content of 5.4%.

**Figure 3:**
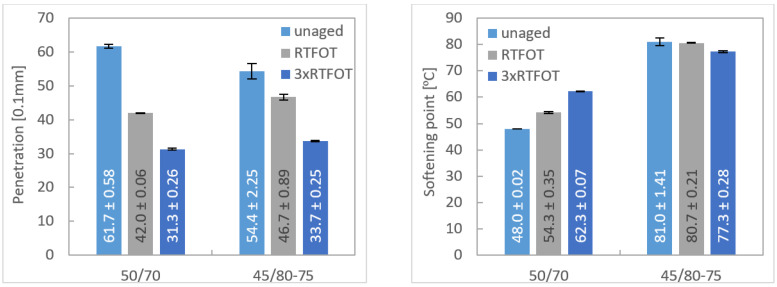
Penetration and softening point of unaged and short-term and long-term aged bitumens.

Two bitumen binders were used to prepare asphalt mixtures and test specimens to investigate their influence on aging. Properties of bituminous binders paving grade bitumen 50/70 and polymer-modified bitumen PMB 45/80-75 are listed in Table 4 and after exposing short and long-term aging in Figure 3. Bitumen aging caused an increase in consistency at intermediate and elevated service temperatures. Penetration values of bitumen decreased by one gradation in the case of paving grade bitumen significantly already after short-term aging and in the case of PMB 45/80-75 after long-term aging. The modified bitumen proved to be more stable in terms of softening point, no significant change occurred. 

**Figure 4:**
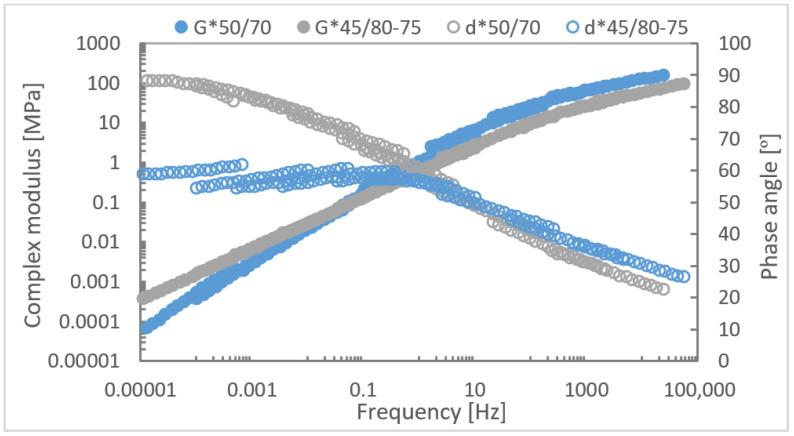
The master curve of tested bitumens.

As the influence of environmental ageing of asphalts was evaluated by the stiffness and indirect tensile strength parameters, the characteristic of the used bitumens was followed by the determination of functional characteristics. The rheological properties of the bitumen were determined using a dynamic shear rheometer over a range of frequencies (0.1 to 10 Hz) and temperatures (−5 to + 60 °C) occurring in the asphalt pavement (master curve calculated for a temperature of 25 °C, Figure 4). The DSR test showed different rheological behaviour of the tested bitumens. At lower temperatures and higher frequencies, 50/70 bitumen has a higher modulus and at higher temperatures and lower frequencies, the modulus of 50/70 bitumen is lower than the modulus of PMB bitumen. 

## 3. Results and Discussion

**Figure 5:**
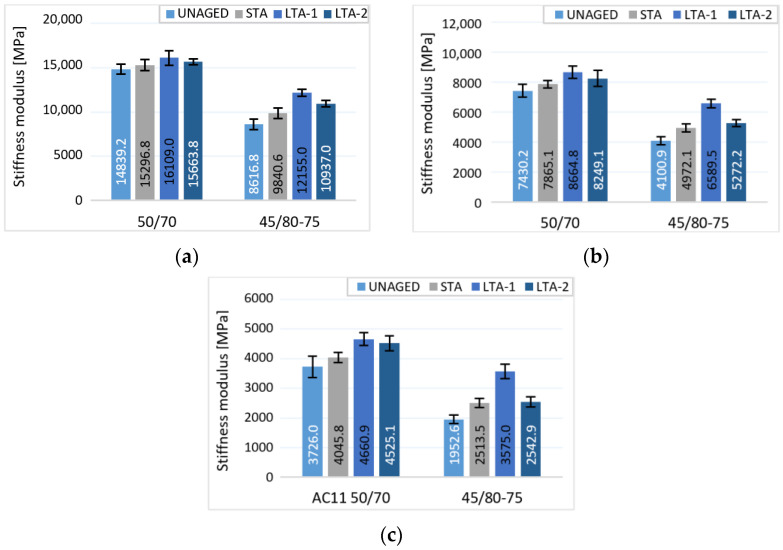
Stiffness of unaged, short-term and long-term aged mixtures AC11 at temperatures (**a**) 10 °C, (**b**) 20 °C and (**c**) 30 °C.

The effect of different stages of ageing of asphalts occurring during the production and transport of the mixture and during the service life after embedment in the pavement was analysed using stiffness. The modulus of stiffness was determined by the IT-CY method for un-aged mixture, short-aged mixture and long-aged mixture specimens by two methods of the loose asphalt mixture and compacted asphalt mixture (Figure 5). The greatest effect of oxidative ageing on asphalt mixture properties was observed for the loose-mixture specimens (LTA-1). This conclusion is confirmed in several studies [33]. Loose mixture ageing is more homogenous ageing with a high oxidation rate. Ageing of compacted samples can change the void content and geometry of the sample. If comparing the tested mixtures, more significant changes in the value of the stiffness modulus occurred for the asphalt mixture with modified bitumen. In the case of loose asphalt mixture, the total area exposed to the effect of thermo-oxidative ageing is greater and affects the whole asphalt mixture. Unlike the compacted specimen, the part of the mixture inside the specimen is not affected by oxidation as in the compacted asphalt layer in the pavement.

The stiffness modulus of the asphalt mixture increases due to aging and partially loses elastic properties, which can be reflected in surface degradation and deterioration. Two fundamental distress modes are cracking and deformation. To ensure the reliability of the pavement structure for the specified requirements and conditions, it is essential to know the mechanical parameters of the pavement materials such as modulus of elasticity, flexural tensile strength, Poisson’s number, stiffness and their variation in time [34]. Empirical methods can be used to an acceptable accuracy to estimate asphalt layer stiffness. Authors in studies [35,36,37] produced dependencies, models and nomographs for the prediction of the stiffness as a function of the stiffness modulus of bitumen and the percentage volumes of asphalt mixture components.

**Figure 6:**
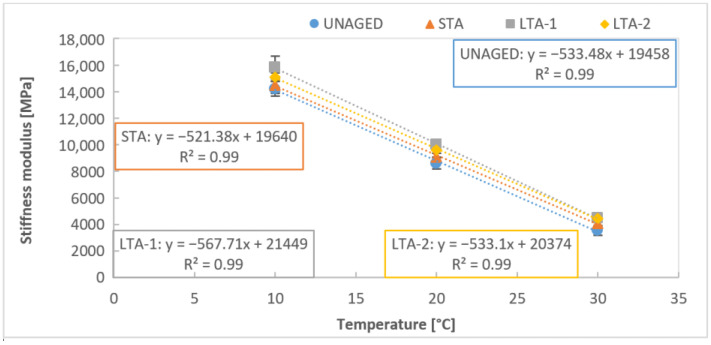
Stiffness of mixture AC11 50/70 unaged, short-term and long-term aged mixtures at different temperatures.

**Figure 7:**
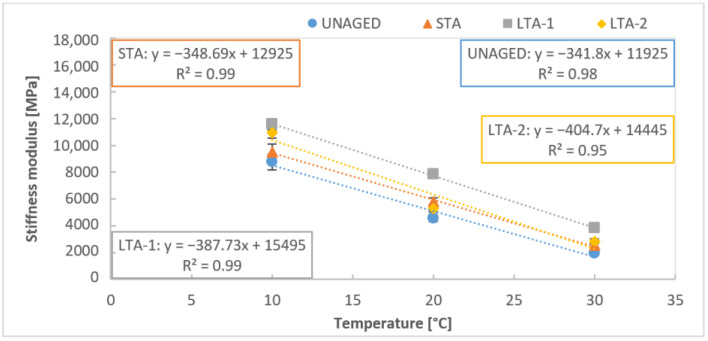
Stiffness of mixture AC11 PMB 45/80-75 unaged, short-term, long-term aged mixtures at different temperatures.

In the study, we did not focus directly on the expression of the dependence of the stiffness modulus on the temperature as several studies [38,39] have already been carried out for this purpose. The results of asphalt stiffness modulus for different temperature dependencies were expressed in this study by a linear function and showed a very similar slope for the asphalts with 50/70 bitumen (Figure 6). This could indicate that the different aging stage (unaged, STA, LTA-1 and LTA-2) does not affect the rate of stiffness change with temperature for the asphalt mixture with 50/70. Figure 7 shows the same dependence of the stiffness modulus of asphalt mixtures with PMB 45/80-75 bitumen of varying temperatures at different stages of aging. The slopes of the linear function are different for different ageing stages. A lower change in stiffness modulus with varying temperatures was observed in the case of the unaged and STA asphalt mixtures. For both methods of long-term aging, the slope is higher and the decrease in stiffness modulus due to temperature change is more pronounced. This indicates that asphalt with PMB 45/80-75 bitumen is more susceptible to ageing, as the aging degree increases the mixture’s sensitivity to temperature increase.

Statistical analysis of the results of asphalt modulus stiffness showed that the influence of ageing is statistically significant for mixtures where the PMB45/80-75 bitumen and specimens produced with 50/70 at higher temperatures (result in statistically significant *p* < 0.05) were used. In comparison at the temperature of 10 °C, there is no statistically significant difference between the mean stiffness values of the tested asphalt specimens with 50/70 at different aging stages (see Table 5). Two different long-term aging methods (LTA-1 loose mixture method and LTA-2 compacted specimen method) were compared. The effect of the long-term aging method was found to be statistically relevant for the mixture with 45/80-75 bitumen. Generally, greater differences in stiffness modulus values are seen due to aging (Figure 5). For the asphalt mixture with 50/70, this effect was not as significantly observed. When comparing the asphalt mixtures in terms of the effect of bitumen type on the stiffness modulus values at different aging stages, the two-factor analysis showed that there was a statistically significant difference between the mean values of the stiffness modulus at all three test temperatures.

**Figure 8:**
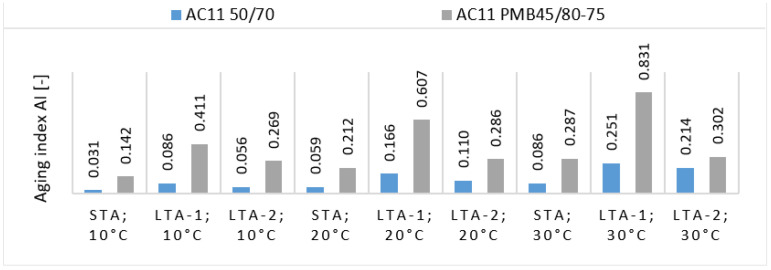
Ageing index AI of tested asphalt mixtures as the ratio of stiffness increase due to ageing.

The properties change rate of asphalt with and without an ageing process is used to quantitatively characterize the ageing degree of asphalt as the ageing index [40,41]. The aging index AI was defined as the ratio of the stiffness modulus of the aged mixture to that of the unaged mixture or alternatively as the ratio of the stiffness increase due to ageing in the unaged mixture, results in Figure 8.

(1) $$\mathrm{AI}=\frac{{\mathrm{Stiffness}}_{\left(\mathrm{STA};\mathrm{LTA}\text{-}1;\mathrm{LTA}\text{-}2\right)}}{{\mathrm{Stiffness}}_{\left(\mathrm{UNAGED}\right)}}$$

or

(2) $$\mathrm{AI}=\frac{{\mathrm{Stiffness}}_{(\mathrm{STA};\mathrm{LTA}\text{-}1;\mathrm{LTA}\text{-}2)\;}-\;{\mathrm{Stiffness}}_{\left(\mathrm{UNAGED}\right)}}{{\mathrm{Stiffness}}_{\left(\mathrm{UNAGED}\right)}}$$

From Figure 8 it can be seen that the largest changes in the AI index were measured after the LTA-1 method of ageing in the asphalt mixture with a modified binder PMB 45/80-75. It seems that in regard to the AI index, the asphalt mixture with modified binder is more susceptible to property changes after long-term aging by the loose spread mixture method. The AC 11 50/70 mixture has significantly lower AI values for all specimens compared to the modified bitumen mixture.

**Figure 9:**
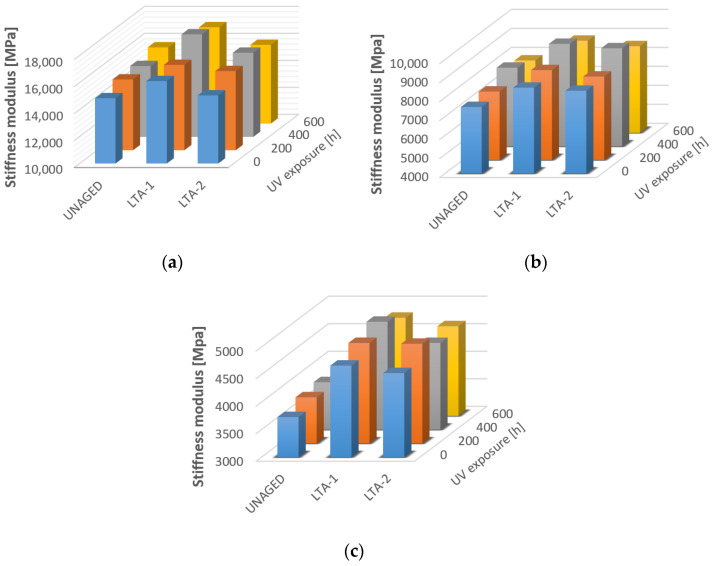
Stiffness modulus of mixtures AC11 50/70 before radiation, after 200, 400 and 600 h of UV radiation at temperatures (**a**) 10 °C, (**b**) 20 °C and (**c**) 30 °C.

**Figure 10:**
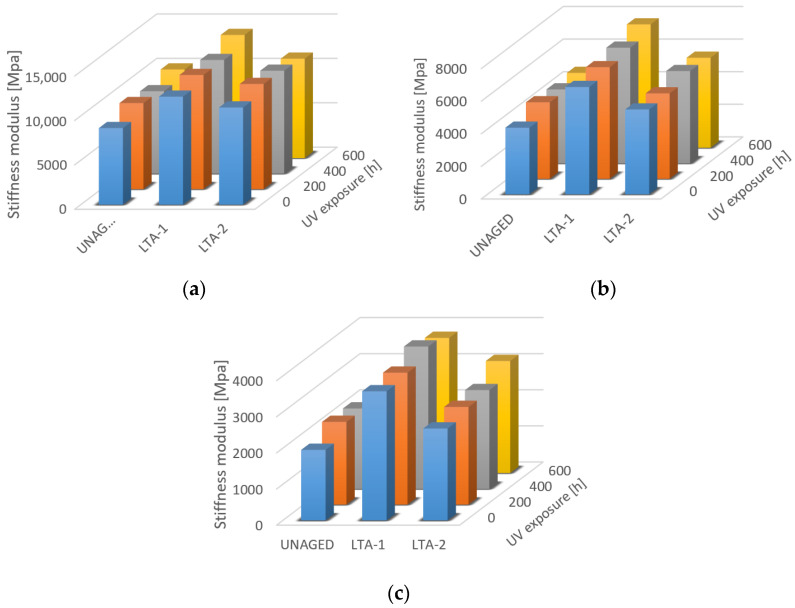
Stiffness modulus of mixtures AC11 PMB 40/80-75 before, after 200, 400 and 600 h of UV radiation at temperatures (**a**) 10 °C, (**b**) 20 °C and (**c**) 30 °C.

Asphalt mixtures in pavement surface layers in service are exposed to solar radiation. Therefore, the UV weathering exposure tests were performed on long-term aged specimens (LTA-1 loos asphalt mixture and LTA-2 compacted asphalt specimen) and unaged asphalts in comparison. The effect of UV radiation on the degradation of the asphalt mixture is demonstrated in Figure 9 and Figure 10. Based on the results of [42], UV radiation exposure was used for weathering effect due to the moisture/condensation reducing the UV photo-oxidative ageing rate.

Figure 9 for asphalt mixtures with 50/70 bitumen showed the increase in the stiffness modulus due to three phases of radiation of 200 h at three different temperatures (10 °C, 20 °C and 30 °C). For all temperatures, a greater or smaller increase in stiffness was observed. The largest differences in stiffness were found at 10 °C, but at the same time, the stiffness values were also the highest at this temperature. In almost all cases, the greatest increase in stiffness of the asphalt mixtures was within the 200–400 h UV phase. In the case of the mixture AC 11 PMB45/80-75 (Figure 10), the highest change due to UV radiation was observed for the unaged specimens. The total increase in stiffness modulus after 600 h of UV exposure was 1557.4 MPa (about 38.1%) at a temperature of 20 °C and 3067.3 MPa (about 35.6%) at 10 °C, and for the AC11 50/70 mixture, the increase was 1055.1 MPa (about 16.7%) at 20 °C and 1623.8 MPa (about 12.4%) at 30 °C.

Previous results have shown the influence of oxidative ageing and UV exposure on the stiffness modulus of the asphalt mixture. Multiple regression analysis was used to test whether certain characteristics significantly predicted the stiffness modulus values of aged asphalts using a correlation analysing model in which the stiffness modulus of short-term aged asphalt, long-term aged asphalt and UV exposure asphalts can be estimated as a linear combination of the unaged asphalt stiffness, the temperature of asphalt, and time of UV exposure. The method of least squares for multiple regression was used to find regression parameters b0, b1, b2, and b3.

The following Table 6 shows the simple linear relationship between the modulus of stiffness of short-term aged, long-term aged asphalt mixtures and UV exposure mixture in the temperature range of 10 to 30 °C and UV radiance exposure range of 0 to 600 h, respectively. The results of the regression indicated the two predictors (stiffness of unaged mixture and temperature) explained more than 97.3% of the variance (adjusted R2, *p* < 0.05, see Table 6). It was found that the regression model was a significantly good fit and the stiffness modulus of the unaged mixture and temperature predicted stiffness modulus after short-term and long-term ageing. Similar results were obtained when samples exposed to UV radiation were analysed (Table 6).

**Figure 11:**
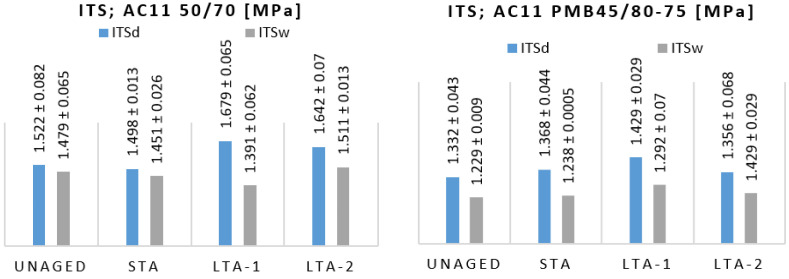
Indirect tensile strength values of tested unaged and aged mixtures, dry and by water effect.

To evaluate the effect of oxidative aging and water on the indirect tensile strength, unaged and long-term aged specimens with higher values of air void content in an average of 6.5 ± 0.98% as verified in [35] were measured. The results (Figure 11) indicate an increase in indirect tensile strengths due to oven conditioning at elevated temperatures with the highest values of the LTA-1 aged asphalts conditioning by the loos mixture method. The accelerated water conditioning influence reduces the indirect tensile strength of asphalt specimens by an average of 7.8% and 8.7% for AC11 50/70 and AC11 PMB 45/80-75 specimens, respectively, with the highest values for LTA-1 loose mix aging of 17.2% and 9.6%, respectively. Statistical analysis Anova (single and two-factor, Table 7) has shown no statistically significant differences between the mean values of the indirect tensile strengths of the specimens with different degrees of aging, and in the case of the tested mixture AC11 PMB 45/80-75, neither of dry nor wet specimens.

**Figure 12:**
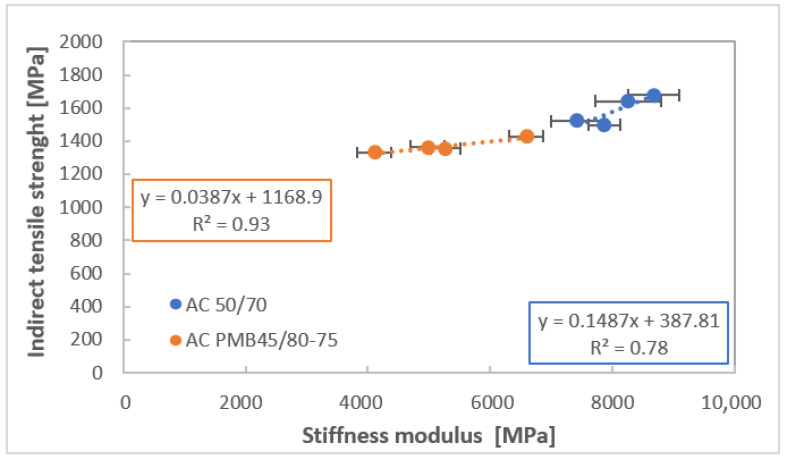
Relation between a stiffness modulus and indirect tension strength at temperature 20 °C.

By comparing the relationship between stiffness modulus applying indirect tension and indirect tensile strength of cylindrical specimens of asphalt mixtures at a temperature of 20 °C (Figure 12), the aging caused more change in the tensile strength of AC 50/70 samples. The stiffness modulus of PMB mixtures are more changed due to ageing as compared to strength. 

## 4. Conclusions

To understand the development characteristics of asphalt properties with the ageing time proceeded by conducting the ageing process of asphalt by way of laboratory simulation, it will make it possible to determine the predicted properties of the mixture during service of the road pavement. The ageing methods were developed by linking to the real situation in the plants during laying and service life. The effect of thermo-oxidative aging, UV radiations and water effects on asphalt properties were determined on asphalt concrete with a maximum aggregate size of 11 mm, bitumen 50/70 and PMB45/80-75. The following conclusions are drawn from the experimental measuring and analysis:

- thermo-oxidative ageing affects the asphalt stiffness modulus, the stiffness modulus increases with increasing aging intensity,
- the ageing effect is statistically significant for mixtures with the PMB45/80-75 bitumen used (*p*-value < 0.001),
- loose mixture ageing (with greater total area exposed to the effect) is more homogenous aging with high oxidation rate for both tested asphalts, although the results of long-term ageing of the 50/70 mixture by both methods were very similar,
- exposure to UV radiation changed the stiffness modulus of asphalt with 50/70 by an average of 13.7% and asphalt with PMB45/80-75 by 27.4%, out of a total of 600 h of UV exposure, the greatest change occurred after the second 200 h of exposure for the asphalt 50/70 and after the first 200 h of exposure for the asphalt PMB45/80-75,
- accelerated water conditioning reduces the indirect tensile strength of asphalt specimens by an average of 7.8% and 8.7% for AC11 50/70 and AC11 PMB 45/80-75, respectively, with the highest values for LTA-1 loose mixture ageing of 17.2% and 9.6%, respectively.
- statistical analysis has shown no statistically significant differences between the mean values of the indirect tensile strengths of AC11 PMB 45/80-75 specimens with different degrees of aging.

All these conclusions are only limited to the materials used in this study and they may be different for other materials (such as SMA mixture, different compositions in terms of aggregate type and bitumen content that are decisive for asphalt layers’ durability). Continued research and measurements of other asphalt properties reflected the real field performance as resistance to permanent deformation, fatigue evaluation and low-temperature cracking should lead to the design and use the asphalt mixtures providing optimal service performance and durability in target application areas.

## Figures and Tables

**Table 1:** Design of research procedure.

Asphalt Mixture	AC 11 50/70; AC 11 PMB 45/80-75	
	IT-CY at 10, 20, 30 °C	ITS_d_, ITS_w_ at 20 °C
Unaged	×	×
STA	×	×
LTA-1 (loose mixture)	×	×
LTA-2 (compacted specimen)	×	×
Unaged+UV (200, 400, 600 h)	×	-
LTA-1+UV (200, 400, 600 h)	×	-
LTA-2+UV (200, 400, 600 h)	×	-

**Table 2:** Ultraviolet spectral power distribution specification for Xenon Arc.

Spectral Bandpass Wavelength λ (nm)	Minimum Percent	Benchmark Solar Radiation Percent	Maximum Percent
λ < 290			0.15
290 ≤ λ ≤ 320	2.6	5.8	7.9
320 < λ ≤ 360	28.3	40.4	40.0
360 < λ < 400	54.2	54.2	67.6

**Table 3:** Particle size distribution of aggregate of asphalt mixture.

Sieve Size [mm]	0.063	0.125	0.25	0.5	1	2	4	8	11.2	16
Passing [%]	6.5	8.6	10.5	13.4	21.1	34.7	52.0	78.8	97.1	100

**Table 4:** Properties of used bitumen binders.

Parameter	Test Method	50/70	45/80-75
Penetration at 25 °C (0.1 mm)	EN 1426	61.7	54.4
Softening point (°C)	EN 1427	48	81
Dynamic viscosity at 135 °C (Pa.s) at 165 °C (Pa.s)	EN 13302	575.6 137.8	2236.0 553.7

**Table 5:** Analysis of variance for stiffness modulus of the tested asphalt mixtures before ageing, after short-term and long-term ageing, α = 0.05; and effect of method of long-term aging.

Effect	Test Temperature	AC11 50/70	AC11 PMB45/80-75
		*p*-Value (d_f_ = 15)	
	20 °C	0.009	<0.001
Effect of aging	10 °C	0.225	<0.001
	30 °C	<0.001	<0.001
	20 °C	0.270	<0.001
Effect of LTA method	10 °C	0.209	0.005
	30 °C	0.449	<0.001

**Table 6:** Multiple regression analysis results of the stiffness modulus of STA mixtures, LTA-1 mixtures and LTA-1+UV mixtures.

Ageing Rate	Mixture	Model	Adjusted R2; *p*-Value
STA	AC 50/70	*E*_(*predicted*)_ = 0.701·*E*_(*unaged*)_ − 163.06·*T* + 6263.39	0.99; 4.76 × 10^−15^
	AC PMB	*E*_(*predicted*)_ = 0.872·*E*_(*unaged*)_ − 110.80·*T* + 3773.94	0.98; 4.82 × 10^−12^
LTA-1	AC 50/70	*E*_(*predicted*)_ = 0.415·*E*_(*unaged*)_ − 308.28·*T* + 12,316.62	0.99; 2.52 × 10^−14^
	AC PMB	*E*_(*predicted*)_ = 0.252·*E*_(*unaged*)_ − 339.35·*T* + 12,930.36	0.97; 2.15 × 10^−11^
LTA-1+UV	AC 50/70	*E*_(*predicted*)_ = 0.719·*E*_(*unaged*)_ − 193.87·*T* + 1.07·*UV* + 7407.07	0.99; 2.15 × 10^−19^
	AC PMB	*E*_(*predicted*)_ = 0.816·*E*_(*unaged*)_ − 160.33·*T* + 1.45·*UV* + 6648.82	0.99; 1.12 × 10^−20^

**Table 7:** Analysis of variance for indirect tensile strength, before aging, after short-term and long-term aging, α = 0.05.

Effect	AC11 50/70	AC11 PMB 45/80-75
	*p*-Value	
Effect of aging (ITSd values)	0.109	0.345
Effect of aging (ITSw values)	0.217	0.672
Effect of aging and water	0.046	0.949

## Data Availability

Data available on request.
